# Entrapment of *α*-Amylase in Agar Beads for Biocatalysis of Macromolecular Substrate

**DOI:** 10.1155/2014/936129

**Published:** 2014-09-15

**Authors:** Manu Sharma, Vinay Sharma, Dipak K. Majumdar

**Affiliations:** ^1^Department of Pharmacy, Banasthali University, Bansthali 304022, India; ^2^Department of Bioscience and Biotechnology, Banasthali University, Banasthali 304022, India; ^3^Department of Pharmaceutics, Delhi Institute of Pharmaceutical Sciences and Research, Formerly College of Pharmacy, University of Delhi Pushp Vihar, Sector III, New Delhi 110017, India

## Abstract

Attempts have been made to optimize immobilization parameters, catalytic property, and stability of immobilized *α*-amylase in agar. The work compares natural entrapment efficiency of agar with the ionotropically cross-linked agar hydrogel, with the advantage of easy scale-up and cost and time effectiveness. Beads prepared with 3% (w/v) agar and 75 mM calcium chloride and hardened for 20 minutes were selected for further studies on the basis of entrapment efficiency (80%) and physical stability. Following entrapment, pH and temperature optima of enzyme were shifted from 6 to 6.5 and 50 to 55°C, respectively. Michaelis constant (*K*_*m*_) for both free and entrapped enzymes remained the same (0.83%) suggesting no change in substrate affinity. However, *V*_max⁡_ of entrapped enzyme decreased ~37.5-fold. The midpoint of thermal inactivation for entrapped enzyme increased by 8 ± 1°C implying its higher thermal stability. The entrapped enzyme in calcium agar bead had an *E_a_* value of 27.49 kcal/mol compared to 17.6 kcal/mol for free enzyme indicating increased stability on entrapment. Half-life of enzyme increased ~2.2 times after entrapment in calcium agar at 60°C indicating stabilization of enzyme. The reusability of beads was size dependent. Beads with diameter <710 *μ*m were stable and could be reused for 6 cycles with ~22% loss in activity.

## 1. Introduction

Enzymes have an enormous potential as catalysts in chemical processes in wide range of industries [1]. *α*-Amylases are one of the largest selling industrial enzymes that find use in a wide variety of industrial applications such as production of ethanol, starch liquefaction, detergents, desizing of textile, modified starches, laundering, dye removal and feed preprocessing, and paper recycling [2]. But the largest volume is sold to the starch industry for conversion of starch to maltose in preparation of high fructose corn syrup and ethanol [3].

Amylases are produced by a wide spectrum of organisms, although each source produces biochemical phenotypes that significantly differ in parameters like pH and temperature optima as well as metal ion requirements [4, 5]. Bacterial amylases used in industrial applications are highly thermostable with optimum temperatures greater than 90°C [3, 6]. Such high temperature optima could lead to deleterious change in foods and result in burnt flavors. Further, high temperature optima (near 100°C) means that the enzymes cannot be used at lower temperatures (60°C) with good efficiency. Fungal amylases (source* Aspergillus niger and Aspergillus oryzae* being mesophilic in nature) have the advantage of high catalytic rates at moderate temperatures of 50–60°C without affecting the sensory appeal in starch liquefaction process leading to refined syrups and sweeteners widely used in the food industry. The starch refining industry spends $62.2 million each year on these enzymes [7]. Since the recovery yield and the reusability of free enzymes as industrial catalysts are quite limited, attention has been paid to enzyme immobilization that offers advantages over free enzymes in choice of batch or continuous processes, rapid termination of reactions, controlled product formation, and ease of enzyme removal from the reaction mixture, saves labor and overhead costs, and has adaptability to various engineering design [8]. Furthermore, the interest in immobilized enzymes and their application to bioprocessing, analytical systems, and enzyme therapy has steadily grown in the past decade. Various studies have been carried out on constraining the enzyme on to a solid support thereby immobilizing it and increasing its stability towards temperature [9–11].

A wide variety of supports have been used for immobilization of amylases, most of which modify the enzyme chemically, hampering the enzyme performance. Physical entrapment of alpha-amylase in ionotropically cross-linked biodegradable hydrogels like calcium alginate and kappa-carrageenan beads has been shown to be a relatively easy, rapid, and safe technique [12, 13]. However, both calcium alginate and kappa-carrageenan get ionized in acidic or slightly acidic environment. Thus, beads lose their integrity and reusability of the enzyme is lost. On the other hand, agar is resistant to acidic hydrolysis. Agar is a naturally occurring heterogeneous colloidal polysaccharide complex of agarose and agaropectin having alternating *α*-(1→3) and *β*-(1→4) linkages. Agarose is an alternating copolymer of 3-linked~-D galactopyranose and 4-linked 3,6-anhydro-*α*-galactopyranose units. The structure of agaropectin is not fully known but it is a sulphonated polysaccharide in which galactose as well as uronic acid is partly esterified with sulphuric acid. However, agar has the ability to form gel at salt free condition. Because of ionic nature of polymer (agaropectin), gelation may be influenced by the presence of electrolytes similar to the carrageenan. However, gels prepared in the presence of metallic ion are substantially stronger than those obtained under salt free conditions. Such gels are known as ionotropic hydrogels which are held together by molecular entanglements and/or secondary forces including ionic, H-bonding, or hydrophobic forces [14]. It is the polymer of choice for entrapment of cells and enzymes because of its cost effectiveness and resistance to acidic hydrolysis. Agar also offers a relatively inert aqueous environment within matrix. Keeping the said information in view, attempts have been made to entrap *α*-amylase in agar in order to optimize the immobilization parameters such as agar concentration, calcium chloride concentration, and hardening time on entrapment of enzyme and physical stability of beads. Attempts have also been made to study the catalytic property and stability of the immobilized enzyme compared to those of native enzyme.

## 2. Materials and Methods

### 2.1. Materials

Potassium dihydrogen phosphate, sodium hydroxide, hydrochloric acid, agar, calcium chloride dihydrate (Qualigens Fine Chemicals, Mumbai, India), and soluble starch and 3,5-dinitrosalicylic acid (DNS) (Himedia Laboratories Pvt. Ltd., Mumbai, India) were used as received. Fungal *α*-amylase (source* Aspergillus oryzae*), sodium potassium tartrate, coomassie brilliant blue G, and ortho-phosphoric acid were purchased from S. D. Fine-Chem Ltd., Mumbai, India. All other chemicals and solvents were of analytical grade and were used without further purification. Double-distilled water was used throughout the study.

### 2.2. Enzyme Immobilization

Agar solution (2–4% w/v) was prepared by heating agar powder dispersion in phosphate buffer (pH 6.8) at 90°C and the solution was cooled to 45°C. Required quantity of enzyme (565 mg *α*-amylase in 100 mL of final solution) was dissolved in agar solution. Afterwards, the solution was degassed under vacuum. Enzyme entrapped agar beads were prepared by dropping the molten agar-enzyme solution through a syringe with 20 G × 1.5 in. flat-tip hypodermic needle to a magnetically stirred cool distilled water or calcium chloride solution (40 mL, 50 mM to 100 mM) at a rate of 5 mL/min and the beads were allowed to harden for specific time (10–30 min). Different concentrations of agar and calcium chloride and different hardening times were tried. The beads were collected by decanting distilled water or calcium chloride solution, washed twice with distilled water, and dried to constant weight in a desiccator at room temperature for 48 h. The filtered distilled water or calcium chloride solution and the two washings were collected for protein content estimation.

Immobilization efficiency was defined as follows:

Immobilization efficiency (%) = [(Theoretical enzyme loaded − Amount of enzyme leached in calcium chloride solution and washings)/Theoretical enzyme loading] × 100.

### 2.3. Determination of Protein Content

Protein contents of free enzyme and washings were determined by Bradford method [15] after some modifications. Appropriately diluted one milliliter of enzyme was added to 1 mL of 0.1 M phosphate buffer pH 7.5 followed by addition of 5 mL Bradford's reagent (0.1 g of coomassie brilliant blue G 250 was dissolved in 50 mL of ethanol followed by addition of 100 mL 85% phosphoric acid and volume was made to 1 L) with uniform mixing. The test tubes were kept for 2 min to develop the color completely. The absorbance was read at 595 nm against blank. The protein content of samples was determined from the standard calibration curve of absorbance versus concentration in *μ*g/mL of protein (BSA).

### 2.4. Enzyme Assay

Amylase activity was determined according to the procedure of Bernfeld et al. [16] after some modifications. Appropriately diluted enzyme was added to 1 mL of buffered soluble starch (1%) substrate solution (pH 6.0). After incubation at 50°C for 5 min in a shaking water bath, the reaction was stopped by the addition of 2 mL of 3,5-dinitrosalicylic acid (DNS) reagent (1% DNS in 30% sodium potassium tartrate and 1.6% sodium hydroxide). The tubes were kept in boiling water bath for 10 min to develop the color after which they were cooled. The absorbance was read at 540 nm in a spectrophotometer, after making up the volume to 10 mL. Amylase activity is expressed as mg of maltose liberated in 5 min by 1 mg enzyme under assay conditions. The amount of maltose formed by test samples was calculated using calibration curve of maltose prepared by plotting absorbance versus different concentrations of maltose (mg/mL) in distilled water at 540 nm spectrophotometrically. Similarly, the enzyme activity of immobilized enzyme was determined by the above mentioned procedure by taking the amount of immobilized enzyme equivalent to 1 mg of free alpha-amylase.

### 2.5. Particle Size Measurement

The particle size of 50 gel beads was measured with a gauge type micrometer (0.01 mm least count, Durga Scientific Pvt. Ltd., Vadodara, India) for each group of samples and the mean particle size was determined.

### 2.6. Enzyme Characteristics

#### 2.6.1. Determination of Kinetic Constants

The kinetic constants of native and entrapped *α*-amylase were determined at different concentrations of buffered soluble starch substrate ranging from 0.2% to 2.0% at pH 6.0 and 50°C. The *K*_*m*_ and *V*_max⁡_ of the native and immobilized enzyme were calculated according to Lineweaver-Burk plot [17].

#### 2.6.2. pH Optimum

pH optimum of both entrapped and free enzymes was tested by assaying activity in the pH range of 3.0–8.0. Buffers (0.1 M) used included acetate (pH 3–5.5) and phosphate (pH 6.0–8.0) buffers. Relative activities at the test pH were calculated assuming maximum activity observed as 100% (during the experiment).

#### 2.6.3. Temperature Optimum

Temperature optima of free and entrapped enzymes were determined by assaying activity at pH 6.0, in the temperature range of 0–80°C. The temperature at which maximum activity was observed was taken as 100% and relative activities at different temperatures were calculated.

#### 2.6.4. Thermal Stability of Free and Entrapped Amylase

*Midpoint of Thermal Inactivation.* Both free and entrapped enzyme samples were incubated for 30 min at different temperatures in the range of 20–70°C. After cooling, residual activity was measured at pH 6.0 by transferring suitable amount to the assay mixture. The midpoint of thermal inactivation, where the activity was diminished by 50%, was calculated from the plot of percent residual activity versus temperature. Activity of the unincubated enzyme was taken as 100%.

*Thermal Inactivation Kinetics.* Kinetics of thermal inactivation of free and entrapped enzymes was studied at different temperatures in the range of 50–70°C for 2 h. Enzyme samples were incubated at test temperature and aliquots withdrawn at appropriate time intervals, that is, after every 30 min. It was immediately cooled in an ice bath and residual activity measured at pH 6.0. Activity of unincubated enzyme was taken as 100%. From a semilogarithmic plot of residual activity versus time, the inactivation rate constant, *k*_in_, was calculated. The temperature dependence of *k*_in_ was analyzed from the Arrhenius plot to obtain the inactivation parameters.

### 2.7. Reusability of Entrapped Amylase

Amylase entrapped agar and calcium agar beads were screened through sieve numbers 16 and 22 according to Indian Pharmacopoeia. Beads which passed through sieve 22 were of particle size <710 *μ*m and those which passed through sieve 16 but were retained by sieve 22 were of particle size 0.71–1.4 mm. To test the reusability, these amylase entrapped agar beads of different sizes (<710 *μ*m and 0.71–1.4 mm) were used to assay enzyme activity. Beads were kept in a small wire mesh basket of sieve number 100 and immersed in 100 mL of 1% buffered starch substrate (pH 6), under stirring. After incubation for 5 min, basket containing beads was removed from the reaction mixture and reused after washing thrice with double distilled water. Enzyme activity was determined at pH 6 in the same manner as described for enzyme assay. The decrease in activity for each cycle was determined assuming activity of beads in the first cycle as 100%.

## 3. Results and Discussion

### 3.1. Entrapment of *α*-Amylase

From Table 1, it can be observed that all the process variables like concentration of agar, calcium chloride, and hardening time affect the entrapment efficiency. As the agar solution was added to cool distilled water, the gelation of agar at the interface of agar solution and distilled water takes place followed by further gelation of interior when kept in cool distilled water. On addition of agar solution to calcium chloride, instantaneous interfacial cross-linking takes place followed by a more gradual gelation of the interior and causes loss of enzyme from the beads, which was found to be proportional to the degree of cross-linking. Increase in viscosity with increase in agar concentration may retard the penetration of distilled water or calcium to the interior of the bead, resulting in decreased cross-linking and hence increased entrapment efficiency. Degree of cross-linking increased with increase in calcium chloride concentration and hardening time, and so entrapment efficiency decreased [14]. Since the agar gels are porous in nature, the retention of beads in distilled water increased the leaching of enzyme.

At the lower concentration of agar, calcium chloride, and shorter hardening time, beads get ruptured during decantation and washing of beads. Calcium agar beads prepared with 3% w/v agar and 75 mM calcium chloride and hardened for 20 min were selected for further studies on the basis of its physical stability and entrapment efficiency. The entrapment efficiency of *α*-amylase in calcium agar beads and agar alone was 80.0% and 63.83%, respectively. Thus, calcium agar provides more efficient entrapment of enzyme than agar alone.

### 3.2. Particle Size

From Table 1, it can be observed that, as the concentration of agar was increased, it resulted in increase in % entrapment as well as particle size while increase in calcium chloride as well as hardening time had reduced the same. The bead size is influenced by the opening through which agar is allowed to pass (which was kept constant) and the viscosity of agar solution. As the concentration of calcium chloride and hardening time was increased, it resulted in smaller particle size due to increased cross-linking, although the negative effect of calcium chloride and hardening time was of less magnitude.

### 3.3. Morphology of the Beads

The spherical shape of the beads in the wet state was usually lost after drying, especially for the beads prepared with low concentration of agar. With increase in concentration of agar from 2 to 4% (w/v), the shape of bead was retained to be slightly spherical. The beads prepared with 2% w/v agar were having lesser mechanical strength and they had the tendency to develop a collapsed center which may be because of mass- and heat-transfer phenomena and/or aggregation of agar helical fibers into bundles and squeezing out of some water from the gel. The shape of the optimized beads changed near to a sphere with a collapsed center during the drying process.

### 3.4. Enzyme Characteristics

#### 3.4.1. Kinetics of Free and Entrapped Enzyme

**Figure 1:**
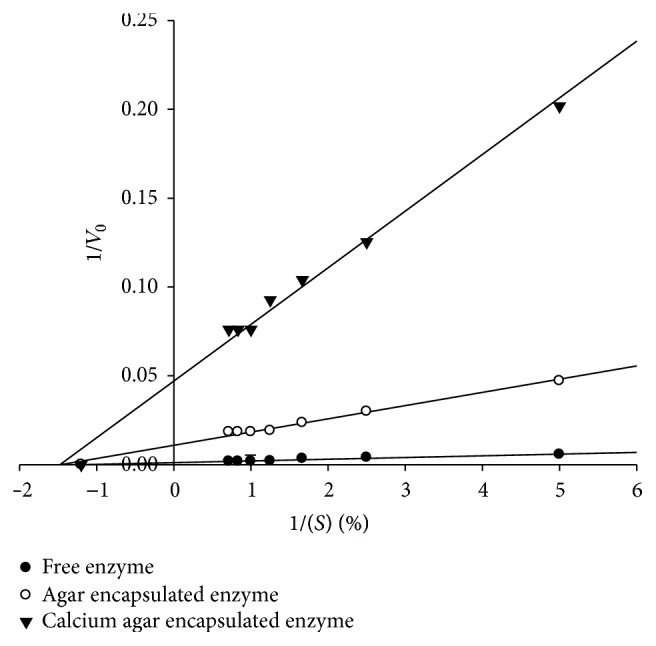
Effect of amylase entrapment on substrate concentration and reaction velocity.

The kinetic parameters, maximum hydrolytic reaction rate (*V*_max⁡_) of enzymatic reaction, and Michaelis-Menten constant (*K*_*m*_) of free and immobilized enzyme were determined by plotting the inverse of hydrolysis reaction rate (*V*_0_) against the inverse of substrate concentration percentage [(S) %] according to the Lineweaver-Burk equation [17]. It was observed that the plot gave a good linear relationship between (1/*V*_0_) and [1/(S %)] as shown in Figure 1. The intercepts of straight line on* y*-axis and* x*-axis corresponded to 1/*V*_max⁡_ and −1/*K*_*m*_, respectively. In enzyme kinetics, *K*_*m*_ is an inverse function of the affinity between enzyme and substrate [16]. Since *K*_*m*_ for free, agar-entrapped, and calcium agar-entrapped enzyme remained the same (0.83%), there is no change in the substrate affinity for the entrapped enzyme compared to free enzyme. Thus, agar entrapment as well as calcium agar entrapment did not have any effect on the binding of substrate to the enzyme. However, *V*_max⁡_ of entrapped enzyme in calcium agar was affected with ~37.5-fold decrease from 833.33 U (for free enzyme) to 22.22 U, while entrapment in agar alone led to decrease in *V*_max⁡_ to 105.26 U, that is, ~8-fold, compared to free enzyme. This indicates that entrapment of the enzyme results in inflexibility of the protein molecule thereby affecting product turnover. Since *V*_max⁡_ is directly proportional to enzyme concentration or number of binding sites, the reduction of *V*_max⁡_ on entrapment suggests reduction in substrate binding sites.

#### 3.4.2. pH and Temperature Optima for Amylase Activity

**Figure 2:**
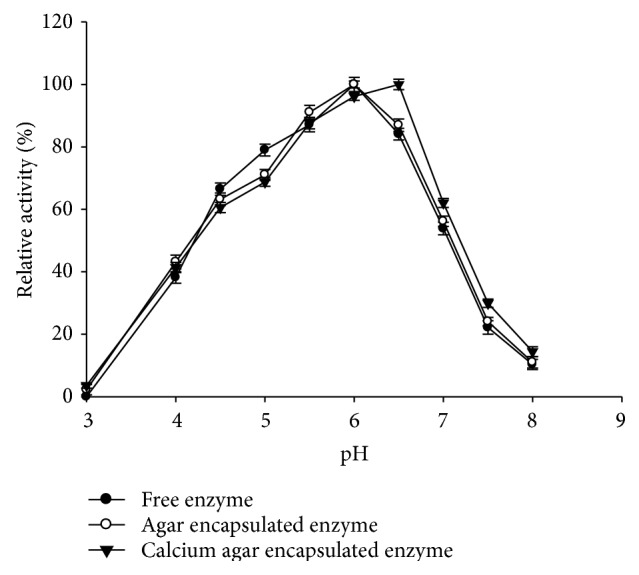
pH optimum of *α*-amylase before and after entrapment in agar and ionotropically cross-linked calcium agar hydrogel.

pH optimum of the free enzyme and enzyme entrapped in agar was 6.0 while pH optimum of the entrapped enzyme in calcium agar was shifted by 0.5 units to 6.5 (Figure 2).

**Figure 3:**
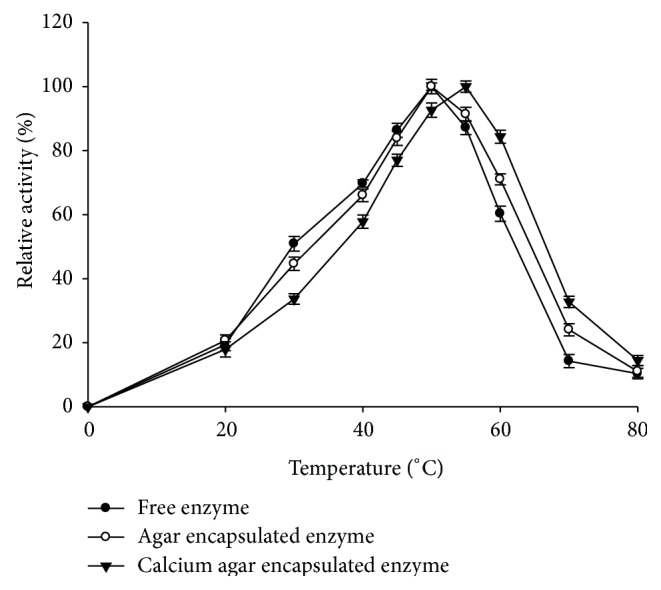
Temperature optimum of free and entrapped *α*-amylase in agar and ionotropically cross-linked calcium agar hydrogel.

The entrapped enzyme in calcium agar was active at a higher temperature range (compared to free enzyme and agar entrapped enzyme) and the optimum temperature for activity was shifted by 5 ± 1°C. The temperature optimum was shifted from 50°C (free and agar entrapped enzyme) to 55°C for the calcium agar entrapped enzyme (Figure 3).

Entrapment affects the enzyme characteristics which are reflected by a change in the pH and temperature optimum of the enzyme. Similar shift in pH and temperature optima on entrapment of *α*-amylase in alginate beads has been reported by Kumar et al. [17]. Higher temperature optima in enzymes are attractive because rate of reactions is faster and there is less microbial contamination of food materials. Kennedy [18] suggested that an increase in temperature optimum can result from a lower temperature in the gel microenvironment compared to bulk solution. Increased thermal stability for immobilized *α*-amylase from* A. oryzae* in polyacrylamide gel has been reported [19].

### 3.5. Thermal Stability of Entrapped Amylase

#### 3.5.1. Midpoint of Thermal Inactivation

**Figure 4:**
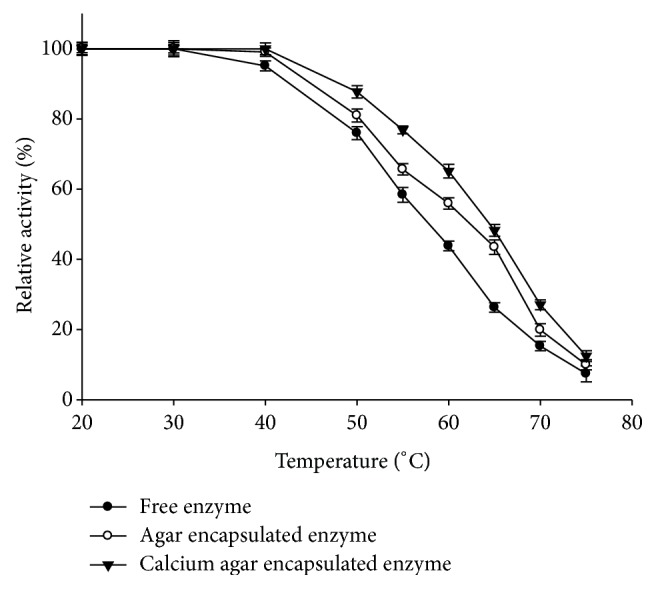
Midpoint of thermal inactivation for free and entrapped *α*-amylase in agar and ionotropically cross-linked calcium agar hydrogel.

Midpoint of thermal inactivation shifted from 57°C (free enzyme) to 61°C and 65°C for the agar entrapped and calcium agar entrapped amylase reflecting increased temperature stability (+8°C) on entrapment in calcium agar (Figure 4).

#### 3.5.2. Thermal Inactivation Parameters

**Figure 5:**
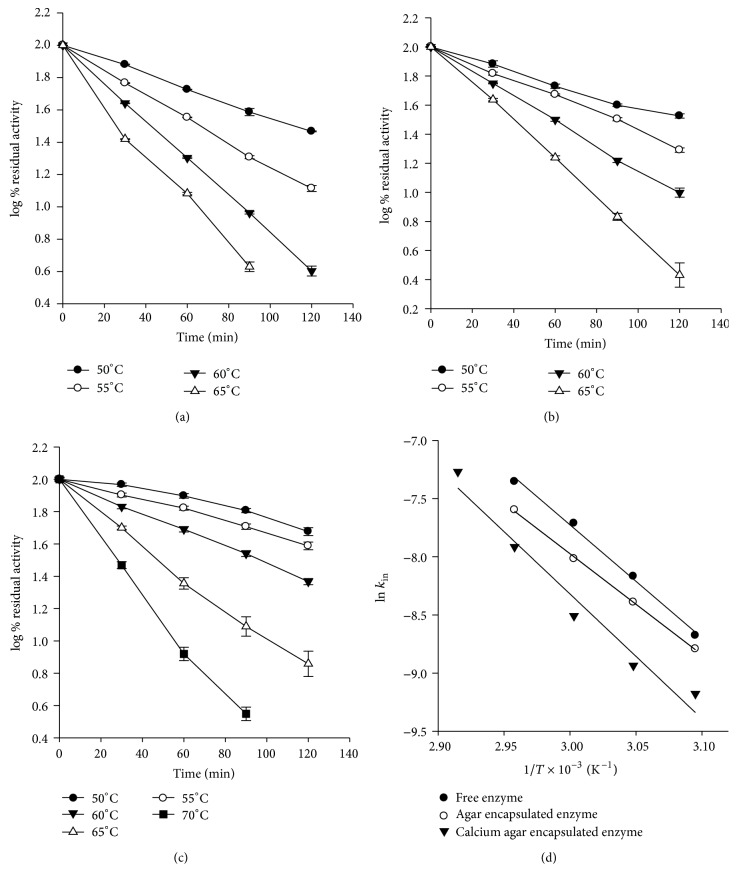
Thermal inactivation kinetics of free and entrapped *α*-amylase. (a) Free enzyme; (b) *α*-amylase entrapped in agar gel; (c) *α*-amylase entrapped in ionotropically complexed calcium agar hydrogel; and (d) Arrhenius plots for free and entrapped enzyme.

Thermal inactivation parameters for the free and entrapped enzyme were determined by incubation at different test temperatures in the range of 50–70°C and assaying for residual activity as a function of time (Figures 5(a), 5(b), and 5(c)). Enzyme inactivation followed first order kinetics. The Arrhenius plot derived from the slopes of the plot of log % residual activity versus time is shown in Figure 5(d).* E*_*a*_ values of entrapped enzyme were higher (27.49 kcal/mol and 20.04 kcal/mol, resp., for enzyme entrapped in calcium agar and agar alone) compared to free enzyme (17.6 kcal/mol). Half-life of the enzyme increased from 25.85 to 57.07 min (~2.2 times higher) after entrapment in calcium agar at 60°C (333 K) indicating stabilization of the enzyme (Table 2).

### 3.6. Reusability of Entrapped Amylase

**Figure 6:**
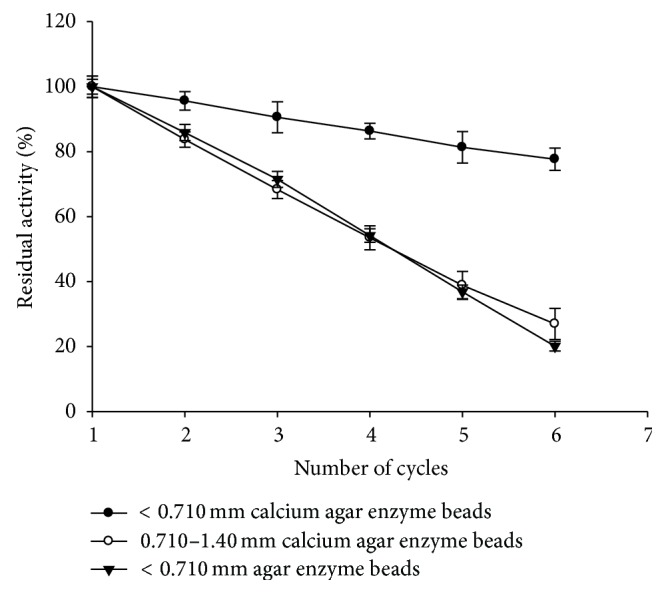
Effect of bead size on the reusability of the entrapped *α*-amylase in calcium agar beads.

The approximate percentage of beads of particle size <710 *μ*m and 0.71–1.40 mm was 85% and 15%, respectively. Operational stability of the entrapped amylase in agar beads and calcium agar beads was evaluated by reusing beads for enzyme assay (after washing with distilled water) for several cycles. The results are shown in Figure 6. Reusability was highly dependent on bead size. Beads with <710 *μ*m diameter were more stable and retained ~78% activity at the end of six cycles for calcium agar beads, while the agar beads of <710 *μ*m diameter retained only 20% activity at the end of sixth cycle. Since the agar beads are porous in nature compared to calcium agar beads, the physical loss of enzyme from the carrier is high which leads to greater decrease in activity compared to calcium agar beads on repetitive use of beads.

Beads with 0.71–1.40 mm diameter lost activity rapidly with <40% residual activity at the end of fifth cycle in case of calcium agar beads. Bead size has a significant effect on the rate of enzymatic reaction [13]. Immobilized enzymes generally experience mass transfer limitations. The size of beads in which *α*-amylase is entrapped may be one of the most important parameters of amylase immobilization. It is expected that enzymes in smaller beads will show higher catalytic activity due to reduced substrate transfer resistance [20]. In the present study, entrapped enzyme in calcium agar beads of <710 *μ*m diameter was more stable and retained ~78% activity at the end of six cycles. Decrease in the enzyme activity during the repetitive use may be due to enzyme denaturation and/or due to physical loss of enzyme from the carrier [13].

Higher half-life represents higher stability which ensures that the enzyme is active for several more cycles compared to less stable enzymes. Immobilized enzymes are more stable compared to free enzymes, due to the restricted rotation and prevention of the unfolding of structure.

Earlier, entrapment of amylase in calcium alginate has been reported [21]. But in the acidic or slightly acidic environment calcium alginate being anionic could break into calcium chloride and alginic acid and beads would lose their integrity and reusability as whole of the entrapped enzyme could leach with complete loss of activity. But amylase entrapped in agar bead is less likely to cause such a problem as the agar is resistant to acidic hydrolysis. So, ionotropically cross-linked agar beads containing *α*-amylase can be used instead of calcium alginate beads if the feed is slightly acidic. *α*-Amylase encapsulation in calcium agar beads thus can serve as a robust and economical technology in the context of food/feed improvement and treatment of biological waste from food processing industries.

## 4. Conclusion

*α*-Amylase from* A. oryzae* was successfully entrapped/immobilized in calcium agar beads with high entrapment efficiency. The entrapped enzyme had higher thermal stability and unchanged substrate affinity and was reusable. The enzyme immobilization in agar is simple, cheap, safe, and easy to scale up. Since the agar is naturally occurring, nontoxic, and biodegradable, the results of the study are useful in the development of a general approach for carbohydrate hydrolysis in food/feed industry and biological waste management.

## Figures and Tables

**Table 1:** Effect of different concentrations of agar, calcium chloride, and hardening time on the percentage entrapment of *α*-amylase and particle size of bead formed.

Agar concentration	Calcium chloride concentration	Hardening time (min)	% entrapment	Particle size (mm)
2%	0 mM	20 min	49.78	0.482 ± 2.92
		30 min	45.35	0.477 ± 1.18
	50 mM	10 min	63.87	0.497 ± 2.21
		20 min	61.32	0.497 ± 1.17
		30 min	58.96	0.485 ± 2.20
	75 mM	10 min	62.50	0.466 ± 1.19
		20 min	57.59	0.461 ± 1.22
		30 min	56.21	0.459 ± 2.18
	100 mM	10 min	55.22	0.460 ± 3.17
		20 min	54.25	0.462 ± 2.15
		30 min	52.86	0.462 ± 2.22

3%	0 mM	20 min	63.83	0.844 ± 3.19
		30 min	61.99	0.842 ± 2.17
	50 mM	10 min	82.68	0.715 ± 2.21
		20 min	78.00	0.708 ± 1.17
		30 min	74.40	0.701 ± 1.20
	75 mM	10 min	82.81	0.682 ± 2.19
		20 min	80.00	0.677 ± 3.22
		30 min	76.26	0.671 ± 3.18
	100 mM	10 min	76.20	0.672 ± 2.21
		20 min	74.68	0.669 ± 1.23
		30 min	73.11	0.663 ± 2.18

4%	0 mM	20 min	64.05	1.12 ± 3.21
		30 min	62.03	1.11 ± 2.19
	50 mM	10 min	78.81	1.02 ± 3.17
		20 min	76.45	1.01 ± 3.20
		30 min	74.88	1.01 ± 2.19
	75 mM	10 min	76.06	0.96 ± 3.22
		20 min	73.51	0.95 ± 2.20
		30 min	71.54	0.95 ± 3.21
	100 mM	10 min	73.11	0.92 ± 3.16
		20 min	70.75	0.91 ± 3.18
		30 min	69.18	0.91 ± 3.21

**Table 2:** Thermodynamic parameters of free and entrapped *α*-amylase.

Temperature (Kelvin)	Free enzyme			Agar entrapped enzyme			Calcium agar entrapped enzyme		
	*k*_in_ × 10^−4^ (s^−1^)	*E*_*a*_ (kcal/mol)	Half-life (min)	*k*_in_ × 10^−4^ (s^−1^)	*E*_*a*_ (kcal/mol)	Half-life (min)	*k*_in_ × 10^−4^ (s^−1^)	*E*_*a*_ (kcal/mol)	Half-life (min)
323	1.71	17.60	67.77	1.52	20.04	76.11	1.03	27.49	111.58
328	2.83	17.60	40.75	2.27	20.04	50.91	1.32	27.49	87.64
333	4.47	17.60	25.85	3.29	20.04	35.06	2.02	27.49	57.07
338	6.40	17.60	18.05	5.02	20.04	23.03	3.65	27.49	31.62
343	—	—	—	—	—	—	6.97	27.49	16.59
